# Supplementary material for: Single-cell profiling reveals differences between human classical adenocarcinoma and mucinous adenocarcinoma
Source: Commun Biol. 2023 Jan 23;6:85. doi: 10.1038/s42003-023-04441-w (PMC9870908; doi:10.1038/s42003-023-04441-w)
Supplement: Supplementary file 2 — Supplementary Information [file 42003_2023_4441_MOESM2_ESM.pdf]

Supplementary Figure 1

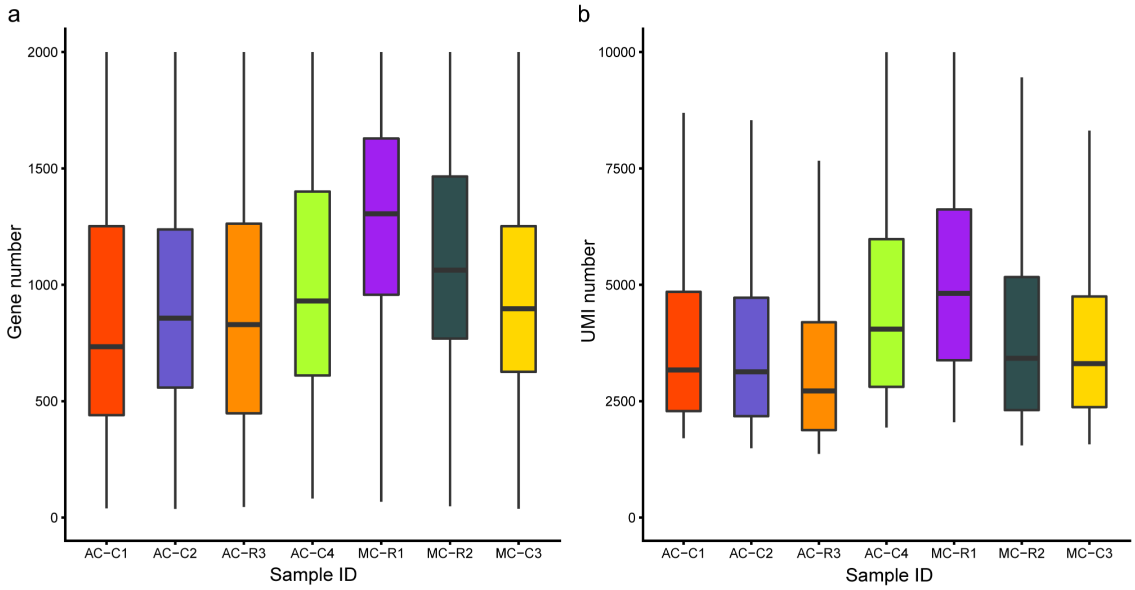

Supplementary Figure 1. Detected gene numbers and UMIs per cell of each patient. a, b The box plot showing the distribution of detected gene numbers **(a)** and UMIs **(b)** per cell in each of the 7 CRC patients. Each box indicates the interquartile range (*IQR*, the range between the 25th and 75th percentile) with mid-point data, and the whiskers represents the upper and lower value within 1.5 times the *IQR*.

Supplementary Figure 2

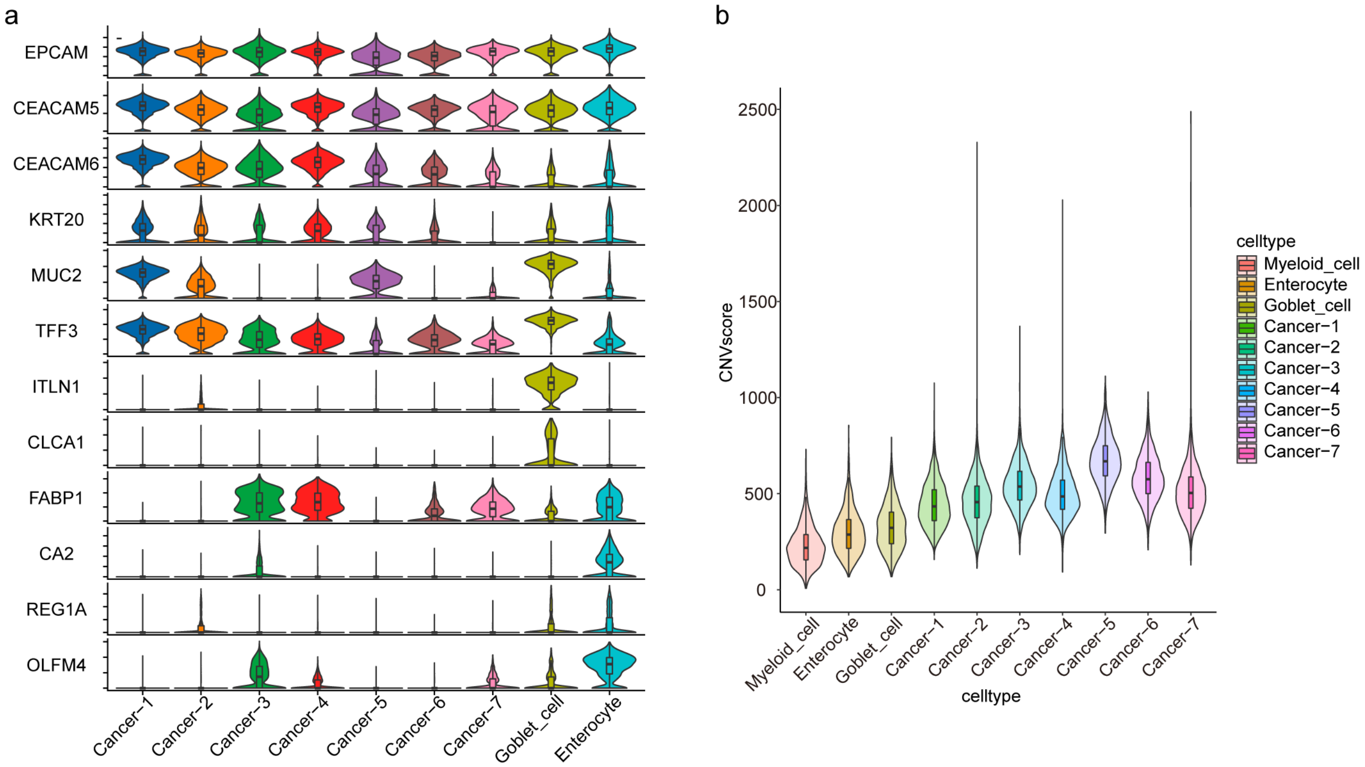

Supplementary Figure 2. Classified cancer cells from the normal epithelial cells. a Violin plots showing the expression distribution of selected marker genes across epithelial cell clusters. **b** Violin plots showing the *CNVscore* of epithelial cell clusters and myeloid cell clusters. The lower hinge, middle line, and upper hinger of boxplots represented the first, second, and third quartiles of the distributions. The upper and lower whiskers corresponded to the largest and smallest data points within the 1.5 interquartile range.

Supplementary Figure 3

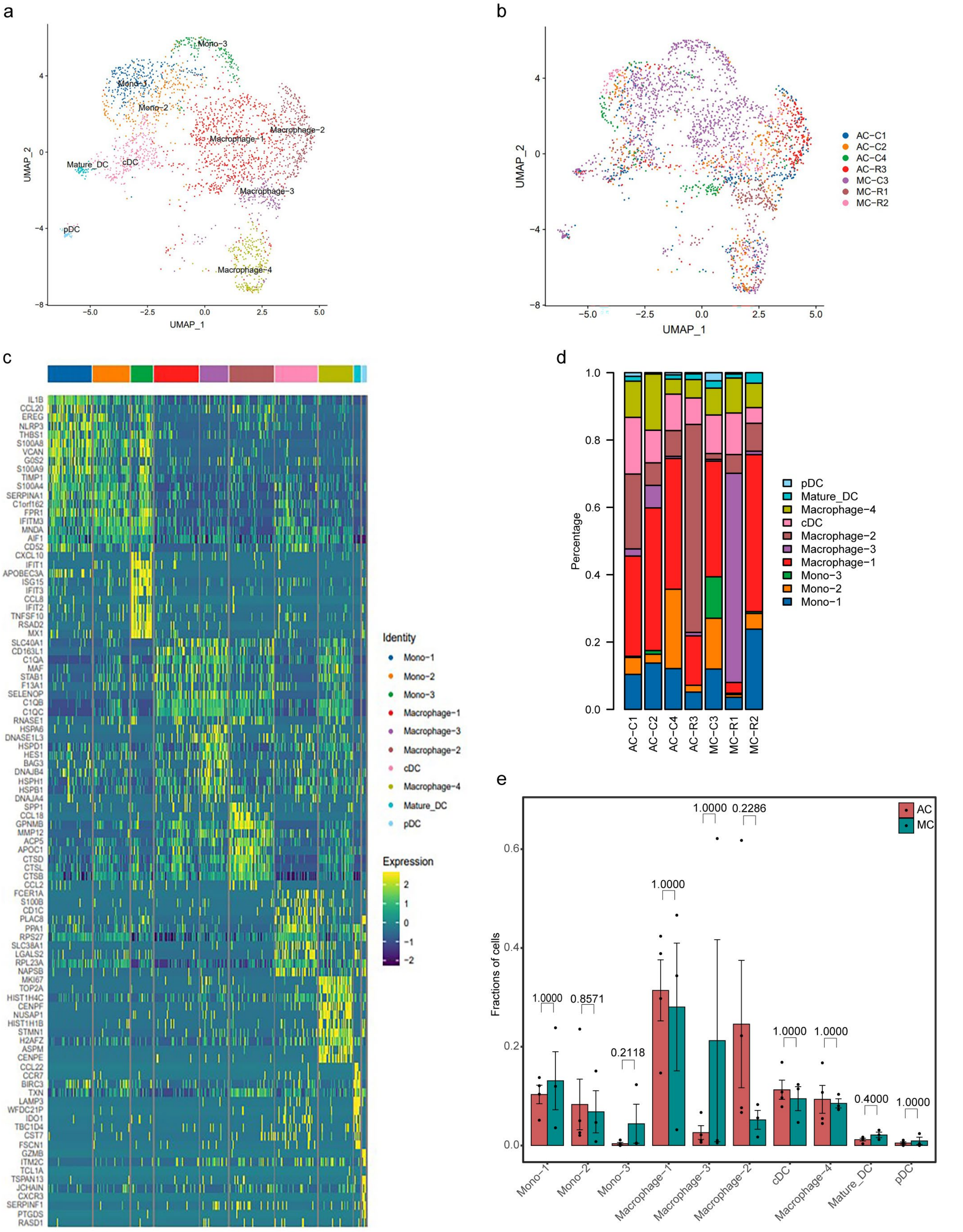

Supplementary Figure 3. Single-cell transcriptomic analysis of Myeloid cells. a UMAP visualization of Myeloid cell clusters. **b** Distribution of 7 samples by UMAP, coloured by patients. **c** Top 10 gene expression in 10 Myeloid cells subclusters. Colour key from blue to yellow represents the scaled expression levels of cell type-specific marker genes from low to high.

d Distribution of the 10 Myeloid cell subclusters among 7 samples. **e** Histogram showing the fraction of Myeloid subclusters in AC (red) and MC (green)**.** The analysis was performed using unpaired two-tailed Wilcoxon rank-sum tests and statistical significance was set at $p < 0.05$. The error bars represent *mean* ± *std*.

Supplementary Figure 4

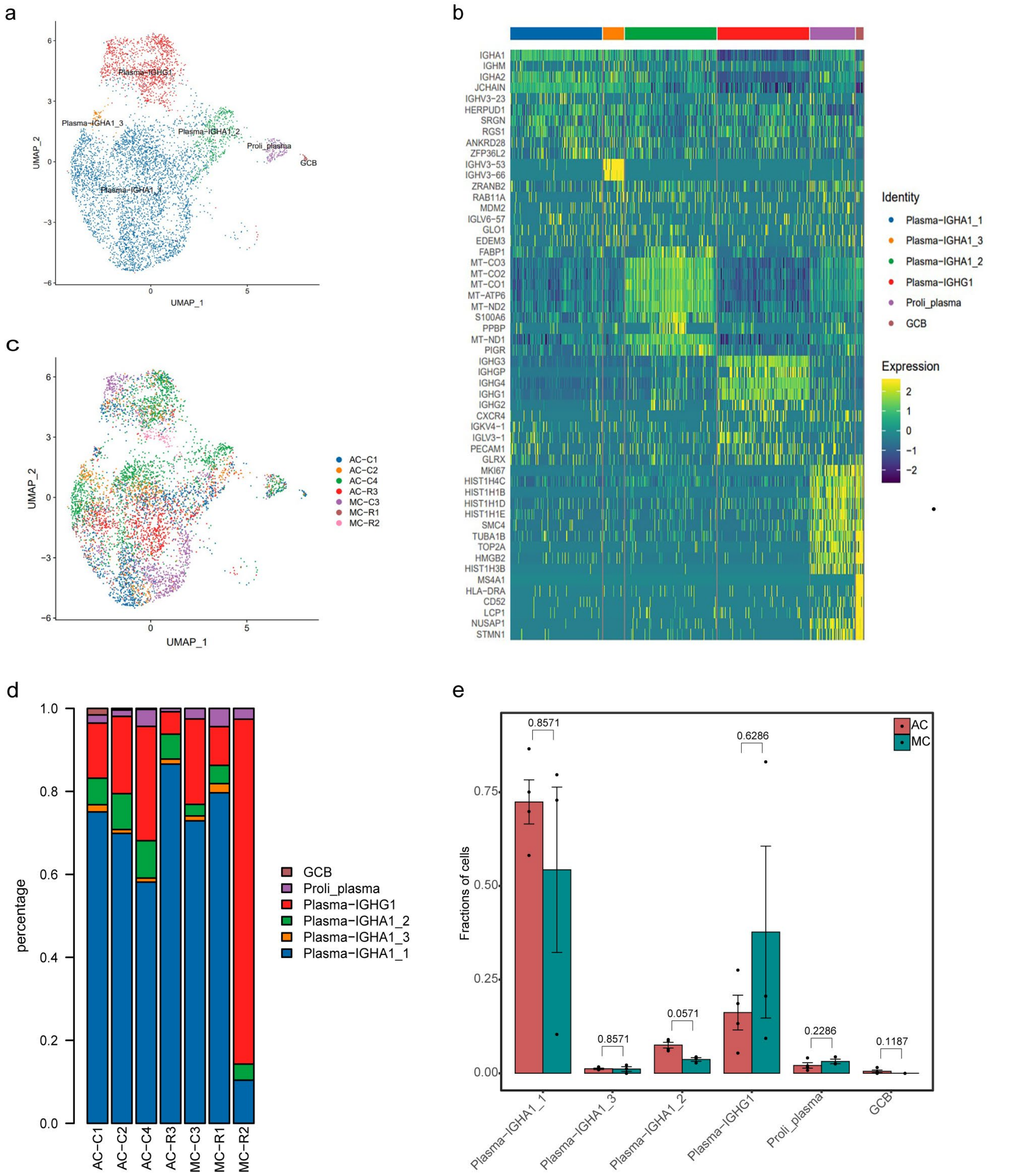

Supplementary Figure 4. Single-cell transcriptomic analysis of B cells. a UMAP visualization of B cell clusters. **b** Top 10

gene expression in 6 B cell subclusters. Colour key from blue to yellow represents the scaled expression levels of cell type-specific marker genes from low to high. **c** Distribution of 7 samples by UMAP, coloured by patients. **d** Distribution of the 6 B cell subclusters among 7 samples. **e** Histogram showing the fraction of B subclusters in AC (red) and MC (green)**.** The analysis was performed using unpaired two-tailed Wilcoxon rank-sum tests and statistical significance was set at $p < 0.05$. The error bars represent *mean* ± *std.*

Supplementary Figure 5

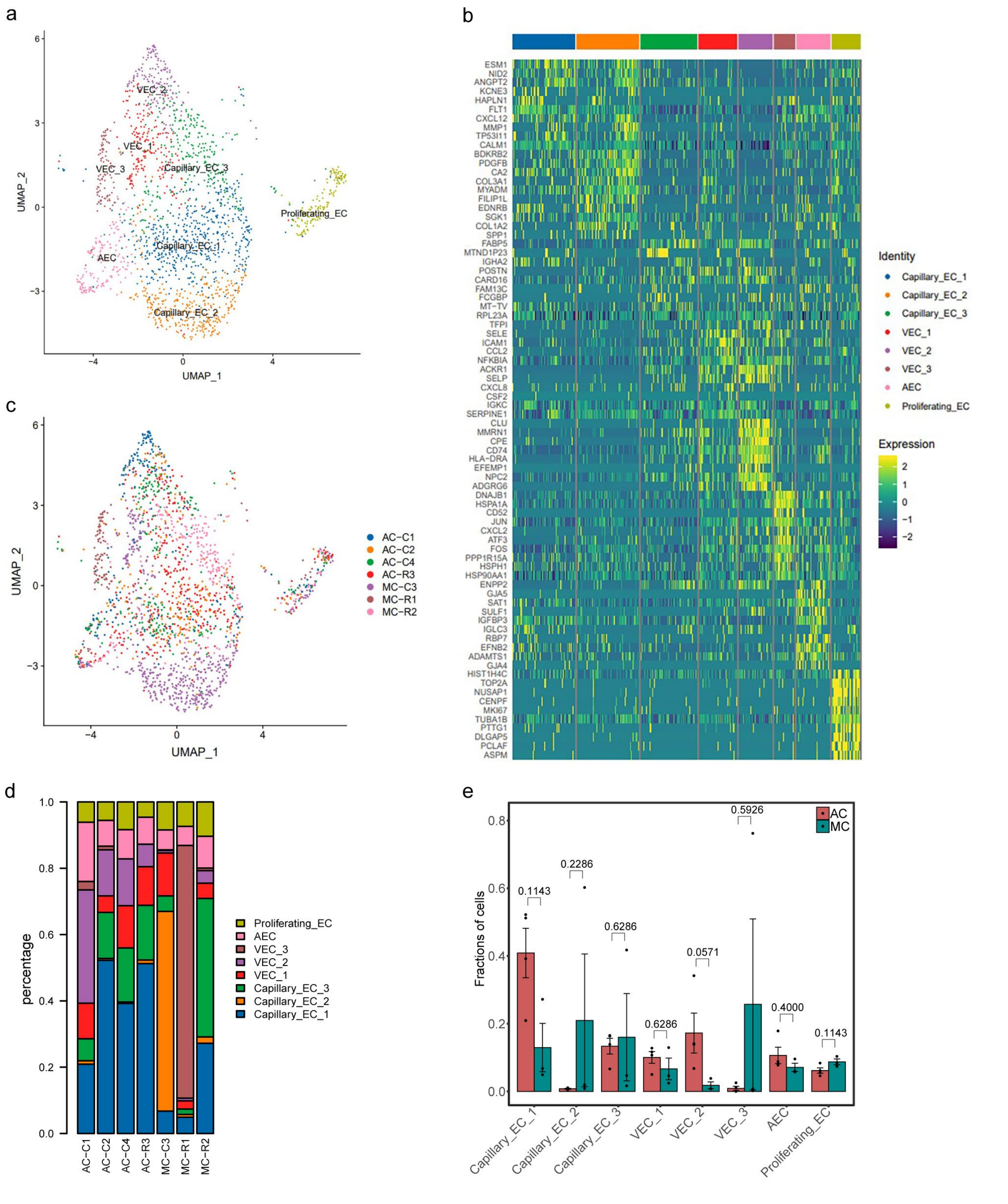

Supplementary Figure 5. Single-cell transcriptomic analysis of endothelial cells. a UMAP visualization of endothelial cell clusters. **b** Distribution of 7 samples by UMAP, coloured by patients. Colour key from blue to yellow represents the scaled expression levels of cell type-specific marker genes from low to high. **c** Top 10 gene expression in 8 endothelial cell subclusters. **d** Distribution of the 8 endothelial cell subclusters among 7 samples. **e** Histogram showing the fraction of endothelial subclusters in AC (red) and MC (green)**.** The analysis was performed using unpaired two-tailed Wilcoxon rank-sum tests and statistical significance was set at $p < 0.05$. The error bars represent *mean* ± *std*.

Supplementary Figure 6

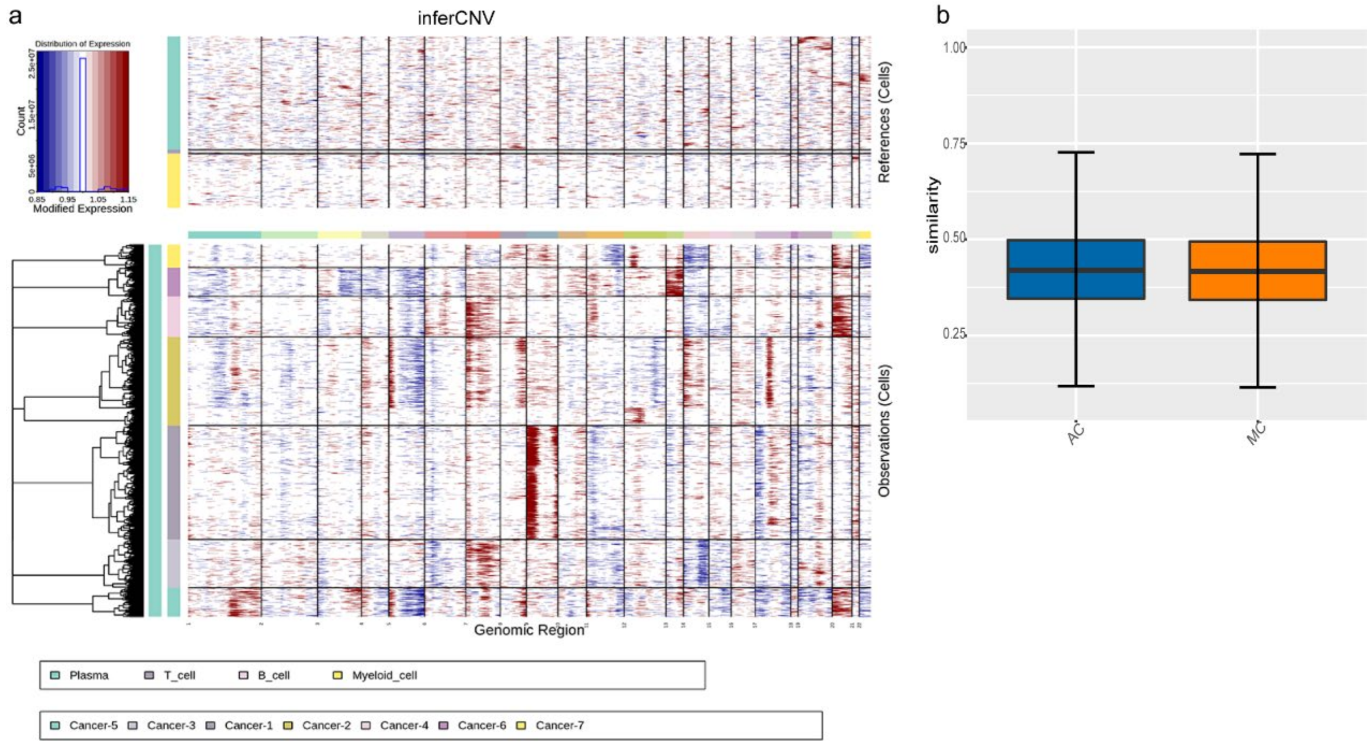

Supplementary Figure 6. Copy-number variation and ITH analysis for CRC cancer cells. a Heatmap of CNV profiles for CRC cancer cells. The CNVs were annotated by the hierarchical clustering to sign the 22 chromosomes according to the inferCNV analysis from each gene expression pattern. Colour key showed the scaled modified *CNV score* of the chromosome of each single cell. Red and blue colours represent high and low CNV level, respectively. **b** Comparison of ITH_{CNA} between AC and MC. The lower hinge, middle line and upper hinger of boxplots represented the first, second and third quartiles of the distributions. The upper and lower whiskers corresponded to the largest and smallest data points within the 1.5 interquartile range.

Supplementary Figure 7

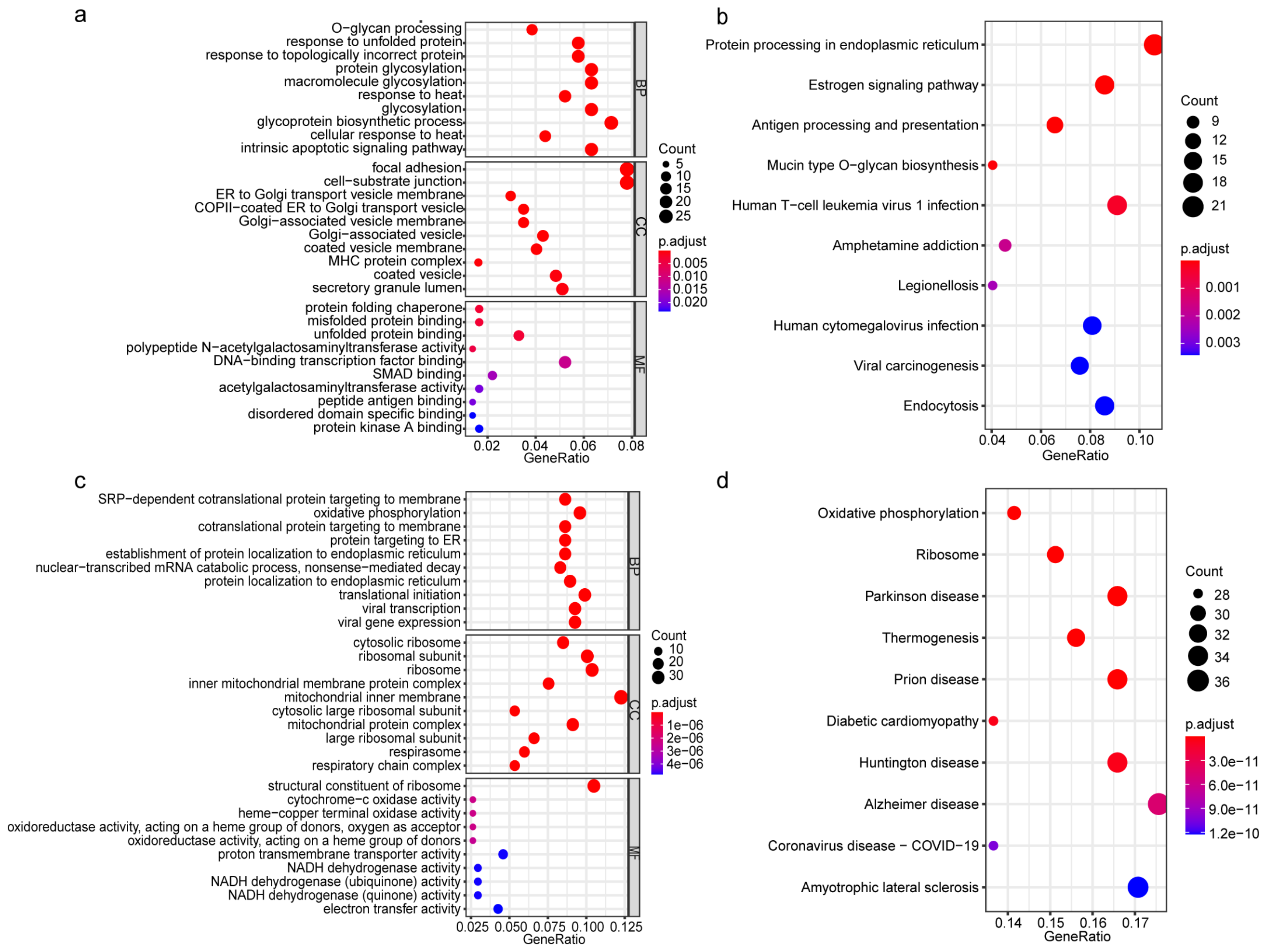

Supplementary Figure 7. a c. Top 10 enriched GO terms for up-regulate and down-regulate genes in MC, respectively. Y axis represents GO terms, and x axis represents rich factor. Size and colour of the bubble represent number of DEGs enriched in GO terms and enrichment significance, respectively. **b d.** Top 10 enriched KEGG pathways for up-regulate and down-regulate genes in MC, respectively. Y axis represents pathway names and x axis represents rich factor. Size and colour of the bubble represent number of DEGs enriched in the pathway and enrichment significance, respectively.

Supplementary Figure 8

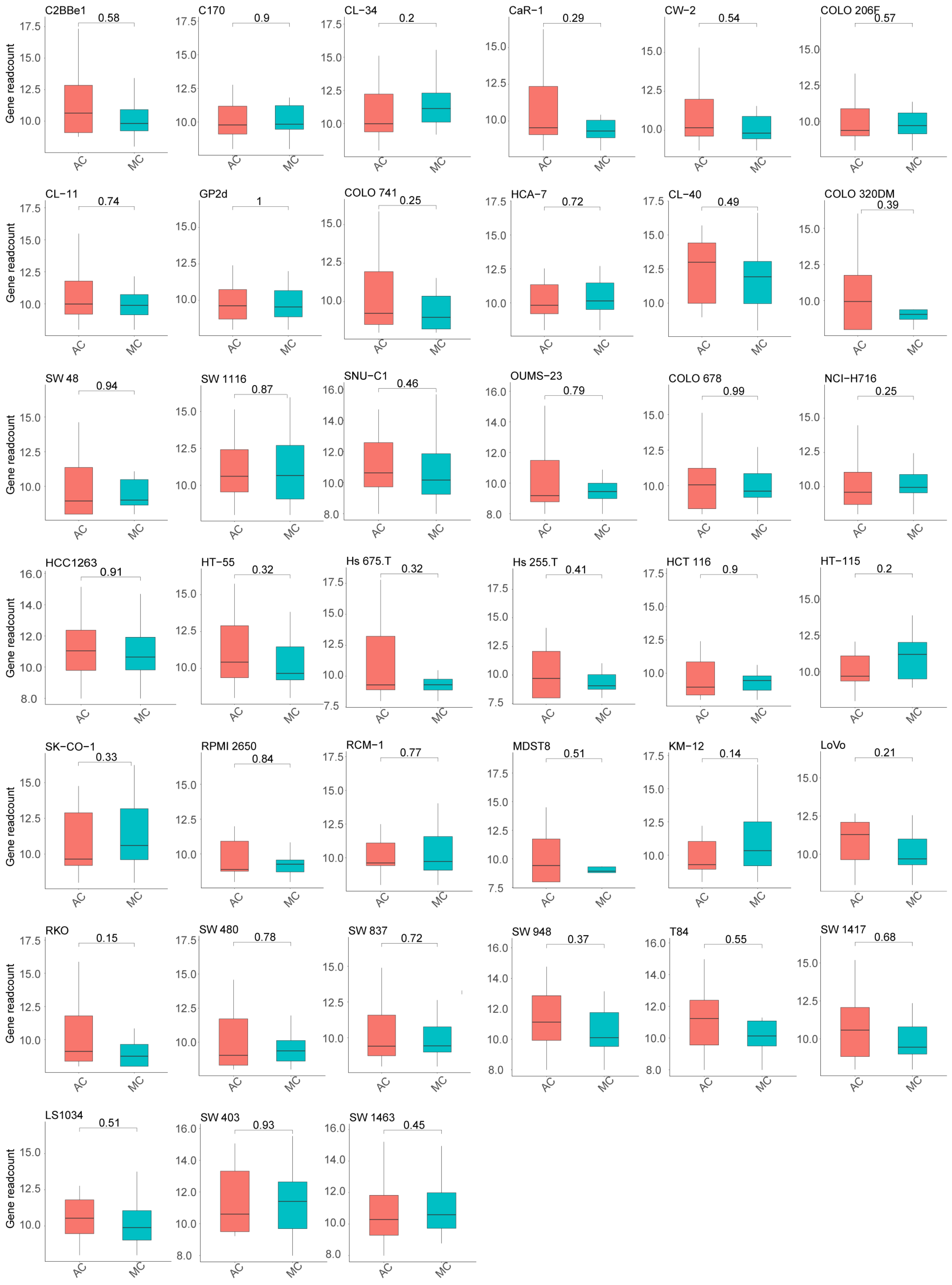

Supplementary Figure 8. Differential expression of MC group or AC group genes in 41 colorectal cancer cell lines.

The analysis was performed using unpaired two-tailed Wilcoxon rank-sum tests, and statistical significance was set at $p < 0.05$. The lower hinge, middle line, and upper hinger of boxplots represented the first, second, and third quartiles of the distributions. The upper and lower whiskers corresponded to the largest and smallest data points within the 1.5 interquartile range.

Supplementary Figure 9

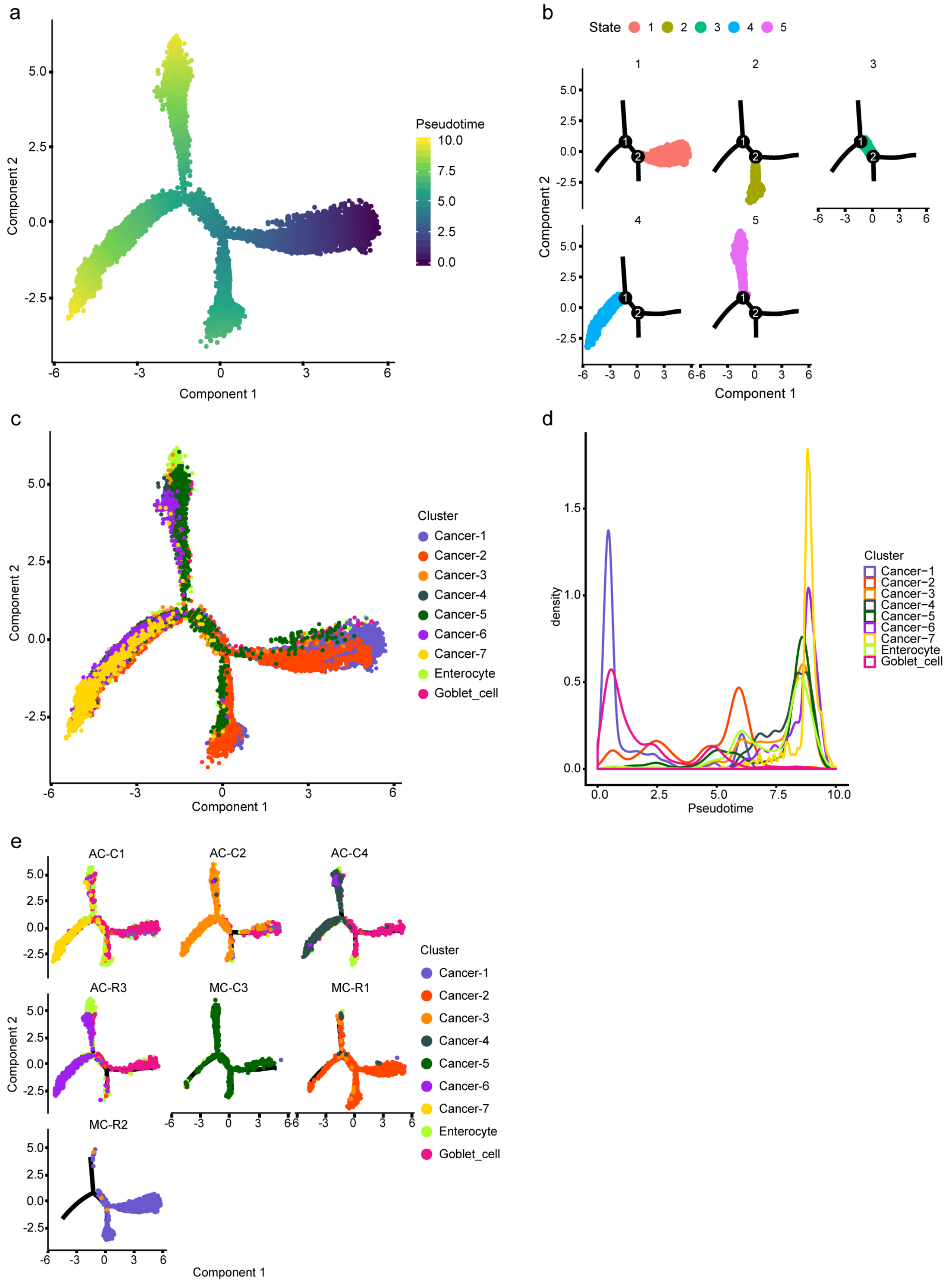

Supplementary Figure 9. The Monocle 2 trajectory analysis of epithelial cells from AC and MC samples. a Pseudo-time ordered analysis of epithelial cells from AC and MC samples. **b** Distribution of cells in different states of the Monocle 2 trajectory plot. **c** The Monocle 2 trajectory plot showing the dynamics of epithelial cell subclusters from AC and MC samples. **d** Cell density distribution by state. **e** Distribution diagram of each sample in the pseudo-time trajectory, different colours indicate the cell types in each sample.

Supplementary Figure 10

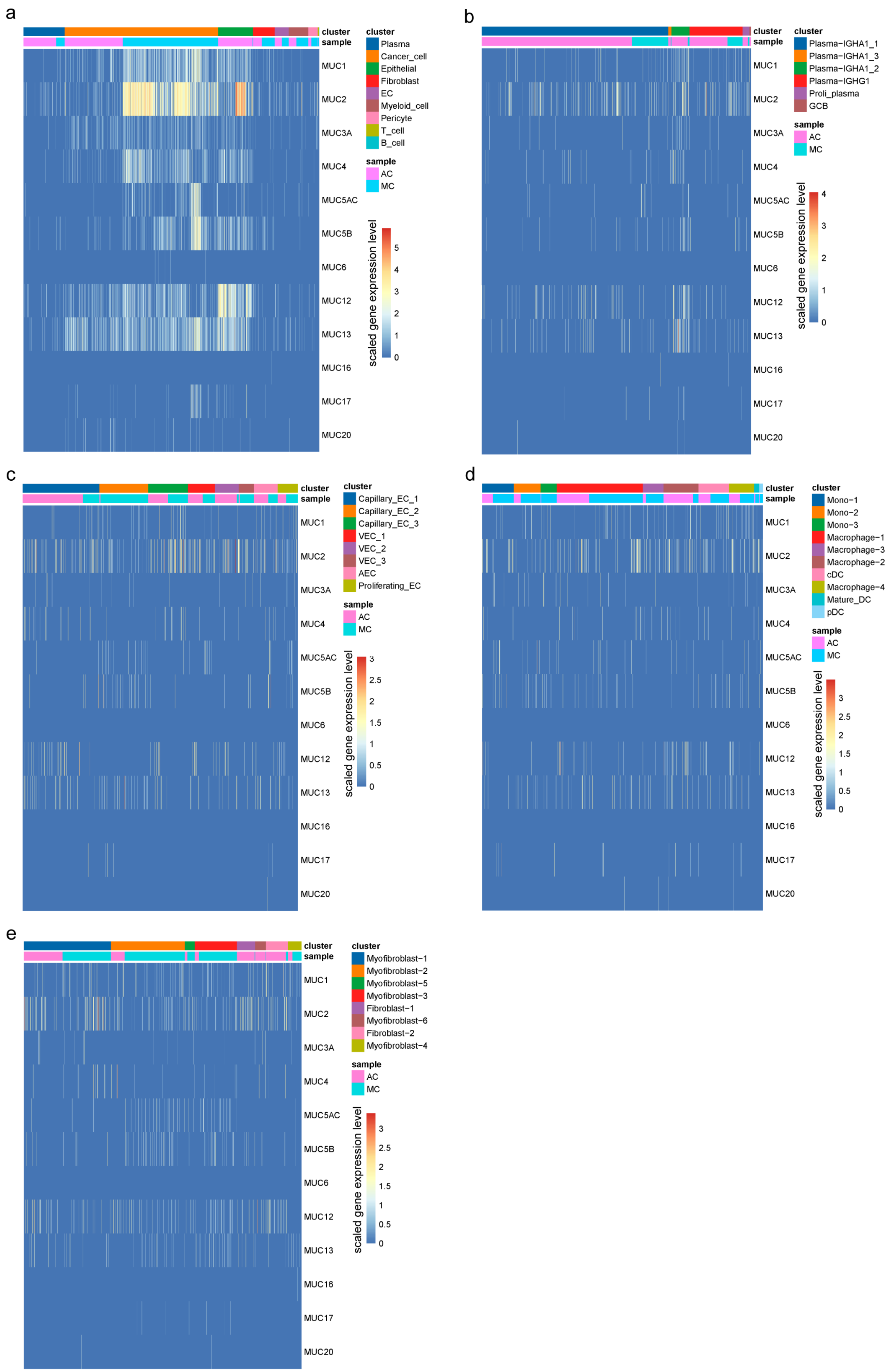

Supplementary Figure 10. MUC family expression profiling at single cell level. a, b, c, d, e Heatmap displaying MUC family expression of 9 main clusters **(a),** B cell subclusters **(b),** endothelial cell subclusters **(c)**, myeloid cell subclusters **(d)**, fibroblast cell subclusters **(e)** in MC and AC.

Supplementary Table 1. Clinical characteristics of the CRC patients.

Sample	Gender	Age	Preoperative treatment	Histology substyle	Location	Size(cm)	Stage
MC-R1	Male	54	Yes	Mucinous adenocarcinoma	Rectal	7.5*6*1.5	IIIC
MC-R2	Female	54	No	Mucinous adenocarcinoma	Rectal	5.8*5.8*1.5	IIIB
MC-C3	Male	52	No	Mucinous adenocarcinoma	Colon ascendens	4*3*2.5	IVC
AC-C1	Male	65	Yes	Adenocarcinoma	Colon sigmoideum	5.5*4.5*0.8	IVA
AC-C2	Female	48	No	Adenocarcinoma	Colon descendens	4*3*0.5	IIIB
AC-R3	Male	39	No	Adenocarcinoma	Rectal	4*3*0.8	IIIB
AC-C4	Male	62	No	Adenocarcinoma	Colon ascendens	4*3.8*1.5	IIA

Supplementary Table 2. The canonical markers for the 9 cell clusters in CRC tissues.

Cell type	Marker genes	Supplementary Reference
Endothelial cells	*PECAM1*, *VWF*	(1) (2)
Pericytes	*RGS5*, *ACTA2*, *PDGFRB*, *MCAM*	(2) (3) (4, 5)
Fibroblasts	*DCN*, *LUM*, *COL1A1*	(6, 7)
Epithelial cells	*EPCAM*, *FABP1*, *CA2*, *MUC2*	(8-11)
Cancer cells	*CEACAM5*, *CEACAM6*, *KRT20*, *MUC2*	(10-13)
T cells	*CD2*, *CD3D/E/G*, *TRAC*, *TRBC1*	(14-18)
B cells	*MS4A1*, *CD79A*, *CD79B*	(8, 14)
Plasma cells	*CD79A*, *JCHAIN*, *MZB1*, *IGHG1*	(4, 7, 19)
Myeloid cells	*LYZ*, *MRC1*, *CD163*, *CD14*, *FCN1*, *CD1C*, *FCER1A*	(4, 16, 19, 20)

Supplementary references

1. Palikuqi B, Nguyen D, Li G, Schreiner R, Pellegata A, Liu Y, et al. Adaptable haemodynamic endothelial cells for organogenesis and tumorigenesis. Nature. 2020;585(7825):426-32.
2. Wang X, Zhou R, Xiong Y, Zhou L, Yan X, Wang M, et al. Sequential fate-switches in stem-like cells drive the tumorigenic trajectory from human neural stem cells to malignant glioma. Cell Res. 2021;31(6):684-702.
3. Corridoni D, Antanaviciute A, Gupta T, Fawkner-Corbett D, Aulicino A, Jagielowicz M, et al. Single-cell atlas of colonic CD8 T cells in ulcerative colitis. Nat Med. 2020;26(9):1480-90.
4. Travaglini K, Nabhan A, Penland L, Sinha R, Gillich A, Sit R, et al. A molecular cell atlas of the human lung from single-cell RNA sequencing. Nature. 2020;587(7835):619-25.
5. Tsukui T, Sun K, Wetter J, Wilson-Kanamori J, Hazelwood L, Henderson N, et al. Collagen-producing lung cell atlas

identifies multiple subsets with distinct localization and relevance to fibrosis. Nature communications. 2020;11(1):1920.
6. Holloway E, Czerwinski M, Tsai Y, Wu J, Wu A, Childs C, et al. Mapping Development of the Human Intestinal Niche at Single-Cell Resolution. Cell stem cell. 2021;28(3):568-80.e4.
7. Habermann A, Gutierrez A, Bui L, Yahn S, Winters N, Calvi C, et al. Single-cell RNA sequencing reveals profibrotic roles of distinct epithelial and mesenchymal lineages in pulmonary fibrosis. Science advances. 2020;6(28):eaba1972.
8. Bao X, Zhang H, Wu W, Cheng S, Dai X, Zhu X, et al. Analysis of the molecular nature associated with microsatellite status in colon cancer identifies clinical implications for immunotherapy. Journal for immunotherapy of cancer. 2020;8(2).
9. Yan H, Siu H, Ho S, Yue S, Gao Y, Tsui W, et al. Organoid cultures of early-onset colorectal cancers reveal distinct and rare genetic profiles. Gut. 2020;69(12):2165-79.
10. Gao S, Yan L, Wang R, Li J, Yong J, Zhou X, et al. Tracing the temporal-spatial transcriptome landscapes of the human fetal digestive tract using single-cell RNA-sequencing. Nat Cell Biol. 2018;20(6):721-34.
11. Parikh K, Antanaviciute A, Fawkner-Corbett D, Jagielowicz M, Aulicino A, Lagerholm C, et al. Colonic epithelial cell diversity in health and inflammatory bowel disease. Nature. 2019;567(7746):49-55.
12. Vieira Braga F, Kar G, Berg M, Carpaij O, Polanski K, Simon L, et al. A cellular census of human lungs identifies novel cell states in health and in asthma. Nat Med. 2019;25(7):1153-63.
13. Chen S, Zhu G, Yang Y, Wang F, Xiao Y, Zhang N, et al. Single-cell analysis reveals transcriptomic remodellings in distinct cell types that contribute to human prostate cancer progression. Nat Cell Biol. 2021;23(1):87-98.
14. Smillie C, Biton M, Ordovas-Montanes J, Sullivan K, Burgin G, Graham D, et al. Intra- and Inter-cellular Rewiring of the Human Colon during Ulcerative Colitis. Cell. 2019;178(3):714-30.e22.
15. Luoma A, Suo S, Williams H, Sharova T, Sullivan K, Manos M, et al. Molecular Pathways of Colon Inflammation Induced by Cancer Immunotherapy. Cell. 2020;182(3):655-71.e22.
16. Li J, Wang R, Zhou X, Wang W, Gao S, Mao Y, et al. Genomic and transcriptomic profiling of carcinogenesis in patients with familial adenomatous polyposis. Gut. 2020;69(7):1283-93.
17. Shami A, Zheng X, Munyoki S, Ma Q, Manske G, Green C, et al. Single-Cell RNA Sequencing of Human, Macaque, and Mouse Testes Uncovers Conserved and Divergent Features of Mammalian Spermatogenesis. Dev Cell. 2020;54(4):529-47.e12.
18. Lambrechts D, Wauters E, Boeckx B, Aibar S, Nittner D, Burton O, et al. Phenotype molding of stromal cells in the lung tumor microenvironment. Nat Med. 2018;24(8):1277-89.
19. Wu F, Fan J, He Y, Xiong A, Yu J, Li Y, et al. Single-cell profiling of tumor heterogeneity and the microenvironment in advanced non-small cell lung cancer. Nature communications. 2021;12(1):2540.
20. Chen E, Chuang L, Giri M, Villaverde N, Hsu N, Sabic K, et al. Inflamed Ulcerative Colitis Regions Associated With MRGPRX2-Mediated Mast Cell Degranulation and Cell Activation Modules, Defining a New Therapeutic Target. Gastroenterology. 2021;160(5):1709-24.
