# Supplementary material for: Single-cell profiling reveals differences between human classical adenocarcinoma and mucinous adenocarcinoma
Source: Commun Biol. 2023 Jan 23;6:85. doi: 10.1038/s42003-023-04441-w (PMC9870908; doi:10.1038/s42003-023-04441-w)
Supplement: Supplementary file 14 — Reporting summary [file 42003_2023_4441_MOESM14_ESM.pdf]

Corresponding author(s): Lin Wang, Ai-Wen Wu

Last updated by author(s): Dec 22, 2022

Reporting Summary

Nature Portfolio wishes to improve the reproducibility of the work that we publish. This form provides structure for consistency and transparency in reporting. For further information on Nature Portfolio policies, see our Editorial Policies and the Editorial Policy Checklist.

Statistics

For all statistical analyses, confirm that the following items are present in the figure legend, table legend, main text, or Methods section.

n/a	Confirmed	
☐	☒	The exact sample size (*n*) for each experimental group/condition, given as a discrete number and unit of measurement
☐	☒	A statement on whether measurements were taken from distinct samples or whether the same sample was measured repeatedly
☐	☒	The statistical test(s) used AND whether they are one- or two-sided<br>*Only common tests should be described solely by name; describe more complex techniques in the Methods section.*
☐	☒	A description of all covariates tested
☐	☒	A description of any assumptions or corrections, such as tests of normality and adjustment for multiple comparisons
☐	☒	A full description of the statistical parameters including central tendency (e.g. means) or other basic estimates (e.g. regression coefficient) AND variation (e.g. standard deviation) or associated estimates of uncertainty (e.g. confidence intervals)
☐	☒	For null hypothesis testing, the test statistic (e.g. *F*, *t*, *r*) with confidence intervals, effect sizes, degrees of freedom and *P* value noted<br>*Give P values as exact values whenever suitable.*
☐	☒	For Bayesian analysis, information on the choice of priors and Markov chain Monte Carlo settings
☐	☒	For hierarchical and complex designs, identification of the appropriate level for tests and full reporting of outcomes
☐	☒	Estimates of effect sizes (e.g. Cohen's *d*, Pearson's *r*), indicating how they were calculated

Our web collection on statistics for biologists contains articles on many of the points above.

Software and code

Policy information about availability of computer code

Data collection	The raw reads were first analyzed using fastQC v0.11.4 and fastp v1.0 to remove low quality of reads, and with cutadapt v1.17 to eliminate poly-A tail and adapter sequences. After extraction of cell barcode and unique molecular identifier (UMI), the reads were mapped to the reference genome GRCh38 (nsemble version 92 annotation) using STAR v2.5.3a. The UMIs and genes of each cell were counted using the featureCounts v1.6.2 software to generate expression matrix files for subsequent analysis.
Data analysis	For data processing and analysis, Seurat (v3.1.2), harmony(v1.0), clusterProfiler (v4.4.4), CellPhoneDB (v2.1.0), Monocle2 (v 2.10.0), pyscenic (v0.11.0) are used under R (v3.6.3) or python (v3.7) for AC and MC cohort.

For manuscripts utilizing custom algorithms or software that are central to the research but not yet described in published literature, software must be made available to editors and reviewers. We strongly encourage code deposition in a community repository (e.g. GitHub). See the Nature Portfolio guidelines for submitting code & software for further information.

Data

Policy information about availability of data

All manuscripts must include a data availability statement. This statement should provide the following information, where applicable:

- Accession codes, unique identifiers, or web links for publicly available datasets
- A description of any restrictions on data availability
- For clinical datasets or third party data, please ensure that the statement adheres to our policy

The original transcriptomic data generated during this study are publicly available in National Genomics Data Center (accession ID: HRA003634). Supplementary Data 4 contains the source data for Figure 2a-b. Supplementary Data 5 contains the source data for Figure 3f. Supplementary Data 8 contains the source data for Figure 5b. The source data that support the findings of Figure 5c are available from GEPIA, http://gepia.cancer-pku.cn/detail.php?gene. Supplementary Data 9 contains the source data for Figure 6b. Supplementary Data 10 contains the source data for Figure 8d. Supplementary Data 11 contains the source data for Figure 9c-d. All other data are available from the corresponding author (or other sources, as applicable) on reasonable request.

Human research participants

Policy information about studies involving human research participants and Sex and Gender in Research.

Reporting on sex and gender

Our findings only apply to gender. Sex and gender were considered in study design and gender was determined based on self-reporting.

Population characteristics

Seven patients (five males and two females, age range from 39 to 65 years old) were diagnosed with nonmucinous adenocarcinoma or mucinous adenocarcinoma according to the consensus standard. Of the patients who provided samples, two had received traditional first-line neoadjuvant chemotherapy, and 5 were naïve patients before surgical therapy. One patient had liver metastasis, and one had omentum metastasis. Detailed clinical characteristics of the patients are provided as follows.

Sample Gender Age Preoperative treatment Histology substyle Location Size(cm） Stage
MC-R1 Male 54 Yes Mucinous adenocarcinoma Rectal 7.5*6*1.5 IIIC
MC-R2 Female 54 No Mucinous adenocarcinoma Rectal 5.8*5.8*1.5 IIIB
MC-C3 Male 52 No Mucinous adenocarcinoma Colon ascendens 4*3*2.5 IVC
AC-C1 Male 65 Yes Adenocarcinoma Colon sigmoideum 5.5*4.5*0.8 IVA
AC-C2 Female 48 No Adenocarcinoma Colon descendens 4*3*0.5 IIIB
AC-R3 Male 39 No Adenocarcinoma Rectal 4*3*0.8 IIIB
AC-C4 Male 62 No Adenocarcinoma Colon ascendens 4*3.8*1.5 IIA

Recruitment

The patient in th hospital who meet a criterion and are volunteer to participate in the study and sign the informed consent. First, we screened colorectal cancer patients with mucus adenocarcinoma and common adenocarcinoma based on pathological results. In addition, we considered gender, age, tumor location, stage and other factors to reduce bias. In addition, two of the sequencing patients received preoperative treatment that may cause genomic changes in tumor and bias the results.

Ethics oversight

The organization has approved the study protocol.

Note that full information on the approval of the study protocol must also be provided in the manuscript.

Field-specific reporting

Please select the one below that is the best fit for your research. If you are not sure, read the appropriate sections before making your selection.

☒ Life sciences ☐ Behavioural & social sciences ☐ Ecological, evolutionary & environmental sciences

For a reference copy of the document with all sections, see nature.com/documents/nr-reporting-summary-flat.pdf

Life sciences study design

All studies must disclose on these points even when the disclosure is negative.

Sample size

For statistical significance, 3 patients were enrolled in the MC group and 4 patients were enrolled in the AC group.

Data exclusions

No data were excluded from the analyses.

Replication

Immunohistochemistry was performed to verify the reproducibility of the experimental findings. All attempts at replication were successful.

Randomization

Randomization is not relevant to our study, because our research is to analyze transcriptional differences of different types of cancer by sequencing.

Blinding	Blinding is not relevant to our study, because we are comparing between groups of known types of cancer.

Reporting for specific materials, systems and methods

We require information from authors about some types of materials, experimental systems and methods used in many studies. Here, indicate whether each material, system or method listed is relevant to your study. If you are not sure if a list item applies to your research, read the appropriate section before selecting a response.

Materials & experimental systems

n/a	Involved in the study	
☐	☒	Antibodies
☒	☐	Eukaryotic cell lines
☒	☐	Palaeontology and archaeology
☒	☐	Animals and other organisms
☒	☐	Clinical data
☒	☐	Dual use research of concern

Methods

n/a	Involved in the study	
☒	☐	ChIP-seq
☒	☐	Flow cytometry
☒	☐	MRI-based neuroimaging

Antibodies

Antibodies used	CEACACAM6 (85102S, CST, USA), REG4 (ab255820, Abcam, UK), FCGBP (ab121202, Abcam, UK), SPINK4 (ab121257, Abcam, UK) and MUC2 (ab134119, Abcam, UK)
Validation	Verification information for CEACACAM6 (85102S, CST, USA) can be found in the manufacturer's website (https://www.cellsignal.com/products/primary-antibodies/ceacam6-e7s7y-rabbit-mab/85102). Verification information for REG4 (ab255820, Abcam, UK) can be found in literature (DOI:10.1371/journal.pone.0109600). Verification information for FCGBP (ab121202, Abcam, UK) can be found in literature (DOI: 10.3892/ol.2021.12787). Verification information for SPINK4 (ab121257, Abcam, UK) can be found in the manufacturer's website (https://www.abcam.com/spink4-antibody-ab121257.html) and literature (DOI:10.1186/s12885-019-6484-5). Verification information for MUC2 (ab134119, Abcam, UK) can be found in the manufacturer's website (https://www.abcam.com/MUC2-antibody-EPR6145-ab134119.html).
